# Factors influencing bamboo intake of captive giant pandas (*Ailuropoda melanoleuca*)

**DOI:** 10.1038/s41598-023-32802-2

**Published:** 2023-04-17

**Authors:** Ming Wei, Yan Zhu, Weiping Liu, Desheng Li, Rongping Wei, Linhua Deng, Kai Wu, Shixian Song, Ti Li, Wen Zeng, Yongguo He, Shan Huang, Chengdong Wang

**Affiliations:** State Forestry and Grassland Administration Key Laboratory of Conservation Biology for Rare Animals of the Giant Panda State Park, China Conservation and Research Center for the Giant Panda, Dujiangyan, 611830 China

**Keywords:** Ecology, Zoology

## Abstract

Bamboo is the main food source of the giant panda. To increase bamboo intake in captive giant pandas, we studied factors affecting the bamboo intake. Fourteen healthy captive giant pandas in Dujiangyan Base of China Conservation and Research Center for The Giant Panda (“Dujiangyan Base” for short) were selected as research objects. A bamboo feeding experiment was conducted to study the effects of seasons, bamboo age, slope orientations where bamboo grows and felling-feeding time on bamboo intake of the giant panda. We found that the type of bamboo that captive giant pandas feed on was abundant in spring and summer, but relatively homogeneous in winter. With the increase of bamboo age, the intake of bamboo leaves decreased, while bamboo culms increased. The feed intake of 1-year-old bamboo leaves and 5-year-old bamboo culms reached the highest respectively. The slope orientation also affected the panda's bamboo intake, and the bamboo growing on sunny slopes or semi-sunny slopes was more favored by captive giant pandas. Moreover, the bamboo intake reached the highest when felling-feeding time was less than 24 h. In short, we confirmed that seasons, bamboo age, slope orientations and felling-feeding time were factors affecting bamboo intake for captive giant pandas. This study was expected to provide scientific guidance improving the feeding behavior management of captive giant pandas.

## Introduction

The giant panda is not only a rare wild animal unique to China, but also a flagship species of biodiversity conservation in the world^1–3^. It has been receiving high attention for many years. The giant panda belongs to carnivorous animals, but it takes bamboo plants as staple food, and it is the only carnivorous species with this special feeding nature^4^. Bamboo is the main source of nutrients for giant pandas. Compared with wild giant pandas who freely choose bamboo as staple food, captive giant pandas are limited by geographical location and environment and can only passively accept bamboo provided by captive units. Therefore, it is extremely important to choose bamboo for captive giant pandas scientifically.

At present, researches on bamboo selection for captive giant pandas have been reported. Fu^5^ and He^6^ found that captive giant pandas in the Qinling Mountains have a wide range of bamboo species selection, and the bamboo selection follows the law of energy economics, that is, they prefer to eat bamboo that can quickly provide the maximum energy, such as bamboo with large leaves and thick mesophyll, or bamboo with tender stems, low lignification and easy to chew. The bamboo species preference of captive giant pandas by Li^7^ and found that captive giant pandas do not like bamboo species with high fiber content. Yang^8^ analyzed the relationship between nutrient content and bamboo intake of captive giant pandas in the Qinling Mountains, and the result indicated that the crude fat, crude protein and ash content of bamboo leaves had a great influence on the selection of staple bamboo species. Meanwhile, the quality of single leaves was also an important factor. Zhao^9^ demonstrated that captive giant pandas tend to choose bamboo with high nutritional value and good palatability in winter. Qu^10^ and Yuan^11^ also confirmed that the bamboo selection of captive giant pandas follows the rule of energy and nutrition, with a bamboo preference for palatability and nutritional value. These researches focused on analyzing the selection preference of captive giant pandas from the perspective of bamboo nutritional composition, but there are few reports on the influence factors of bamboo intake from environment or others.

Studies have shown that in different seasons, the wild panda will eat different bamboo species and different parts of the bamboo, its seasonal feeding changes are the result of its long-term evolution^11^. The captive giant panda, due to the geographical restrictions, mainly rely on artificial supplies of bamboo^12^. Hansen et al. have studied the feeding behavior of two captive giant pandas on bamboo leaves and bamboo culms in different seasons^13^. Therefore, in order to further clarify the influence of seasonal changes on the diet of captive giant pandas, and further optimize the diet composition of captive giant pandas, we studied the bamboo intake of captive giant pandas in different seasons.

The change of bamboo age affects the tannin content in bamboo^14^, and tannin content affects the intake of bamboo by giant pandas. Zhao et al. found that the intake of bamboo by giant pandas increased with the decrease of tannin content^15^. Therefore, we speculate that bamboo age may affect the bamboo intake of captive giant pandas.

Slope orientation is an important topographic factor, which has an important impact on plant growth and development by changing ecological factors such as light, temperature and humidity. Studies have found that slope orientation affects bamboo growth height, carbon storage and bamboo forest density^16–18^. At the same time, combined with more than 20 years of breeding experience, we found that captive giant pandas prefer to eat bamboo growing in the sunny slope, but no relevant reports have been seen so far.

Felling-feeding time refers to the time taken from felling to transporting bamboo to feeding grounds. The length of felling-feeding time affects the moisture content and freshness of bamboo. Based on the long-term observation of the feeding behavior of captive giant pandas, we found that they prefer fresh bamboo. Therefore, we speculated that the length of felling-feeding time may affect the bamboo feeding of captive giant pandas, so as to improve the breeding management of captive giant pandas by optimizing the length of felling-feeding time.

In this study, fourteen healthy captive giant pandas in Dujiangyan Base were selected as research objects to analyze the effects of seasons, bamboo age, slope orientation and felling-feeding time on bamboo intake. These results were expected to provide scientific guidance and reference for enhancing the bamboo intake and improving the feeding behavior management of captive giant pandas.

## Results

### Composition and feed intake of staple food bamboo for captive giant pandas in seasonal variation

**Figure 1:**
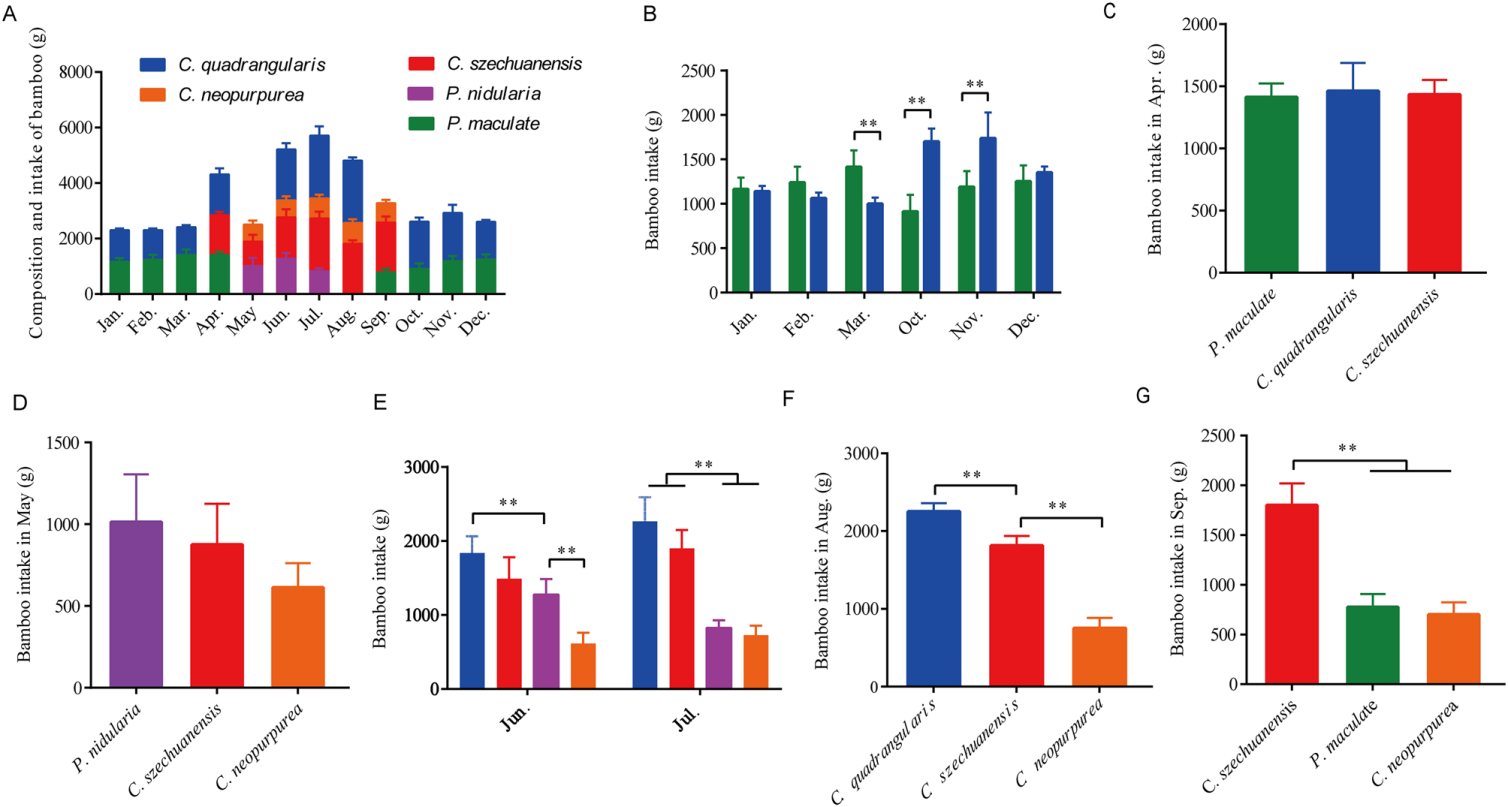
Composition and feed intake of staple food bamboo for captive giant pandas in seasonal variation.

To understand the feed intake of staple food bamboo for captive giant pandas in seasonal variation, we first analyzed the bamboo type in different months, and the result showed that the type of bamboo that captive giant pandas feed on varied in different seasons (Fig. 1A). The type of bamboo in spring and summer (Apr. to Aug.) was abundant, including *Chimonobambusa quadrangularis*, *Chimonobambusa neopurpurea*, *Chimonobambusa szechuanensis* and *Phyllostachys nidularia*, while relatively homogeneous in winter (Nov. to Mar. of the next year), including *C. quadrangularis* and *Pleioblastus maculate*. The bamboo type in autumn (Sep. to Oct.) was in transition between summer and winter. In Sep., the main bamboo species eaten by captive giant pandas were *C. neopurpurea*, *C. szechuanensis* and *P. maculate*, while in Oct., the main bamboo species were *C. quadrangularis* and *P. maculate* (Fig. 1A).

Next, we analyzed the monthly bamboo intake respectively, and the results showed that the intake of *P. maculate* in Mar. was significantly higher than that of *C. quadrangularis* (*p* < 0.01), while the intake of *P. maculate* in Oct. and Nov. was significantly lower than that of *C. quadrangularis* (*p* < 0.01) (Fig. 1B). In Apr., there was no significant difference between feed intake for *P. maculate*, *C. quadrangularis* and *C. szechuanensis* (*p* > 0.05) (Fig. 1C), and in the same way, there was no significant difference in the intake of *C. szechuanensis* and *C. neopurpurea* in May (*p* < 0.05) (Fig. 1D). The intake of *C. quadrangularis* in June was significantly higher than that of *P. nidularia* (*p* < 0.01), and the feed intake of *P. nidularia* was significantly higher than that of *C. neopurpurea* (*p* < 0.01) (Fig. 1E). In August, the feed intake of *C. szechuanensis* and *C. neopurpurea* was significantly lower than that of *C. quadrangularis* (*p* < 0.01) (Fig. 1F). Moreover, the intake of *C. szechuanensis* was higher than that of *P. nidularia* and *C. neopurpurea* (*p* < 0.01) (Fig. 1G). Therefore, the above results indicated that the composition of bamboo species varied in different seasons, and captive giant pandas had different preferences for bamboo in the same season.

### Effects of bamboo age on bamboo intake

**Figure 2:**
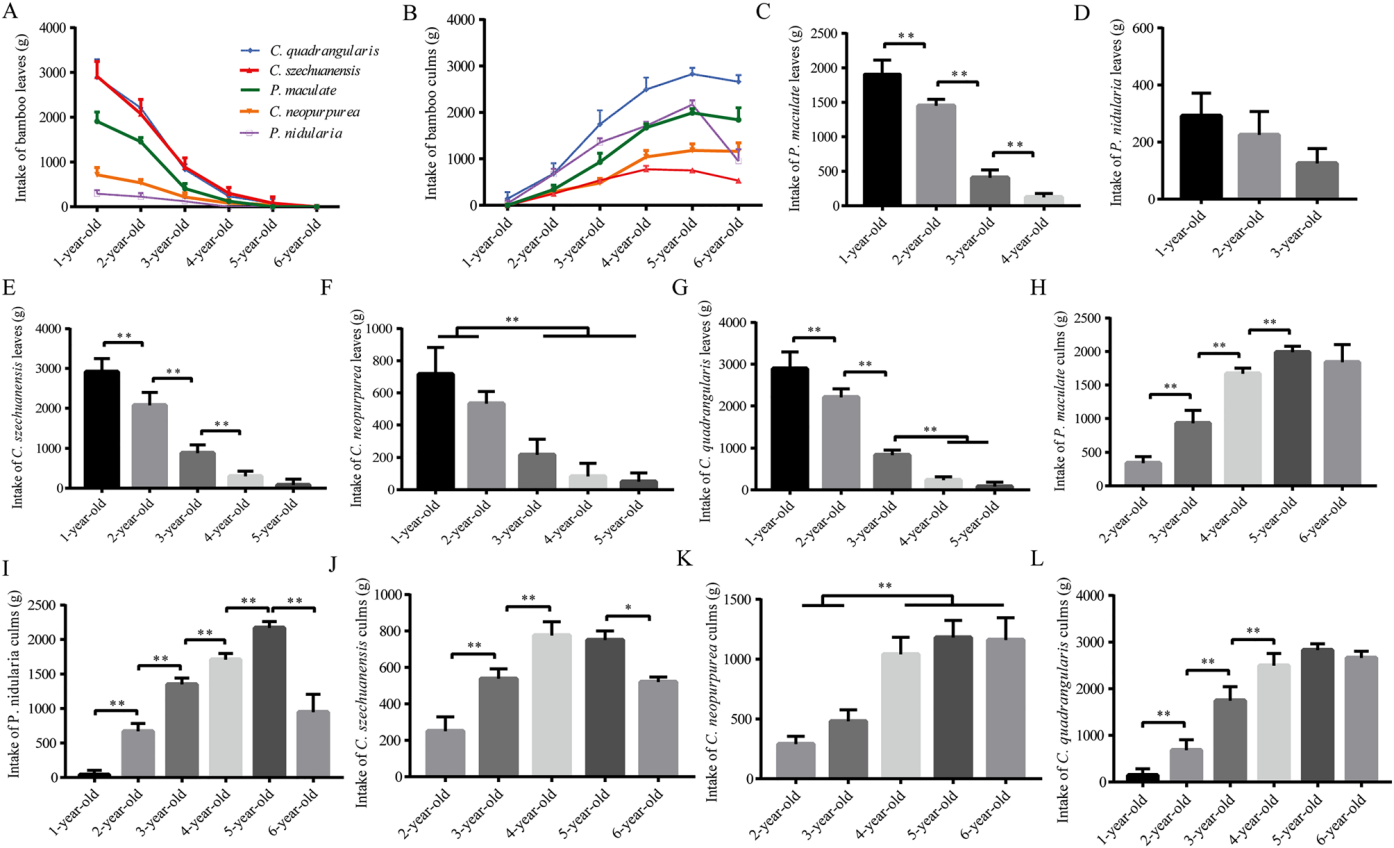
Feed intake of bamboo for captive giant pandas at different bamboo ages.

Fiber content and palatability of bamboo varied with different bamboo ages. To understand the effect of bamboo age on bamboo intake of captive giant pandas, we analyzed the feed intake of bamboo at different bamboo ages under the same conditions of other variables, such as the same bamboo slope orientation and felling-feeding time. Our results showed that the intake of 1-year-old bamboo leaves was the highest for captive giant pandas in the Base (Fig. 2A). The leaves intake decreased with the increase of bamboo age, and the intake of 5-year-old and 6-year-old bamboo leaves was almost zero (Fig. 2A). However, the intake of bamboo culms increased with the increase bamboo age, and the intake of 5-year-old bamboo culms reached the peak (Fig. 2B).

Due to the feed intake of different bamboo species is different in captive giant pandas, we have carried out a detailed study on this. Our results showed that the intake of 1 to 2- year- old bamboo leaves of *P. maculate*, *C. szechuanensis*, *C. neopurpurea and C. quadrangularis* was significantly higher than that of 4 to 6- year- old bamboo leaves (*p* < 0.01) (Fig. 2C,E–G), but there was no significant difference in the intake of bamboo leaves of *P. nidularia* among 1 to 3 years old (*p* > 0.05) (Fig. 2D) (bamboo leaves of 4, 5 and 6 years old were not taken). With the increase of bamboo age, the culm intake of *P. maculate*, *P. nidularia, C. szechuanensis*, *C. neopurpurea and C. quadrangularis* was increased respectively, but there was no rise for the bamboo of 6-year-old (*p* < 0.01) (Fig. 2H–L). The above results revealed that bamboo intake was significantly affected by bamboo age.

### Effects of slope orientation on bamboo intake

**Figure 3:**
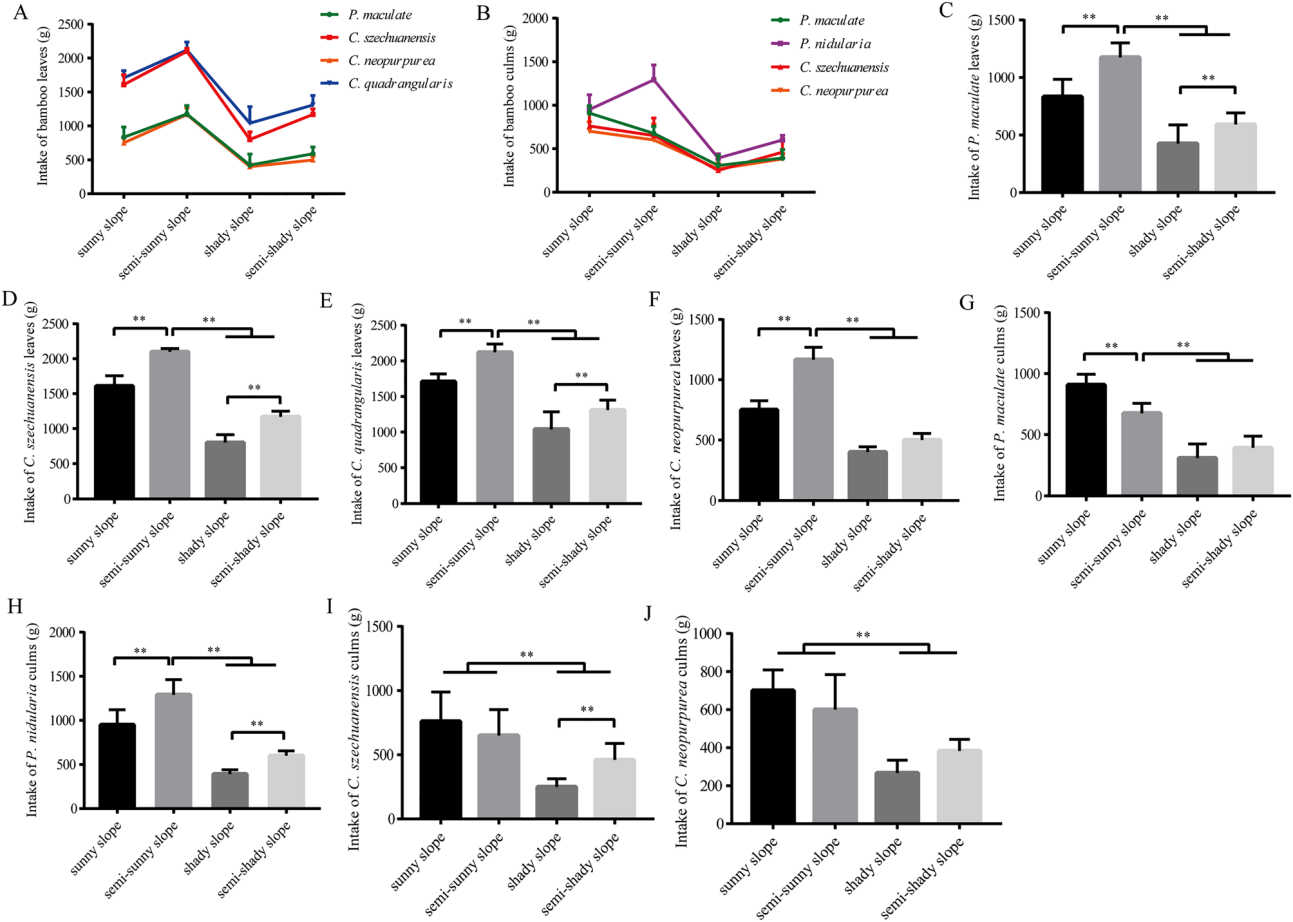
Feed intake of bamboo growing on different slope orientations for captive giant pandas.

Previous studies have shown that there are complex and variable environmental factors such as light, soil moisture, soil temperature and soil mineral content in different slope orientations^19^. To understand the effect of slope orientation on bamboo intake of captive giant pandas, we analyzed the feed intake of bamboo growing on four different slope orientations under the same conditions of other variables, such as the same bamboo age and felling-feeding time. Due to bamboo supply factors, only culms of *P. nidularia* and leaves of *C. quadrangularis* were fed to giant pandas. Our results showed that the order of leaves intake from high to low was semi-sunny slope, sunny slope, semi-shady slope and shady slope (Fig. 3A), and that of culms intake was sunny slope, semi-sun slope, semi-shady slope and shady slope (except *P. nidularia*) (Fig. 3B).

Then feed intake of bamboo leaves and culms in different bamboo species was analyzed respectively, and we found that bamboo intake of leaves of *C. quadrangularis*, *C. szechuanensis*, *P. maculate*, *C. neopurpurea* growing on the semi-sunny slope was significantly higher than that of leaves growing on other slopes, and culms of *P. nidularia* growing on the semi-sunny slope was also significantly higher than that of culms growing on other slopes (*p* < 0.01) (Fig. 3C–F,H). Culms intake of *P. maculate* growing on the sunny slope was significantly higher than that of culms growing on other slopes (*p* < 0.01) (Fig. 3G). There was no significant difference in the feed intake of *C. szechuanensis* culms and *C. neopurpurea* culms between sunny slope and semi-sunny slope (*p* > 0.05) (Fig. 3I,J), but there was a significant difference in the intake of culms between sunny slope and shady slope (Fig. 3I,J) (*p* < 0.01).

Therefore, these results indicated that slope orientation where bamboo grows was one of the factors affecting bamboo intake of captive giant pandas, and the bamboo growing on sunny or semi-sunny slopes was more favored by captive giant pandas.

### Effects of felling-feeding time on bamboo intake

**Figure 4:**
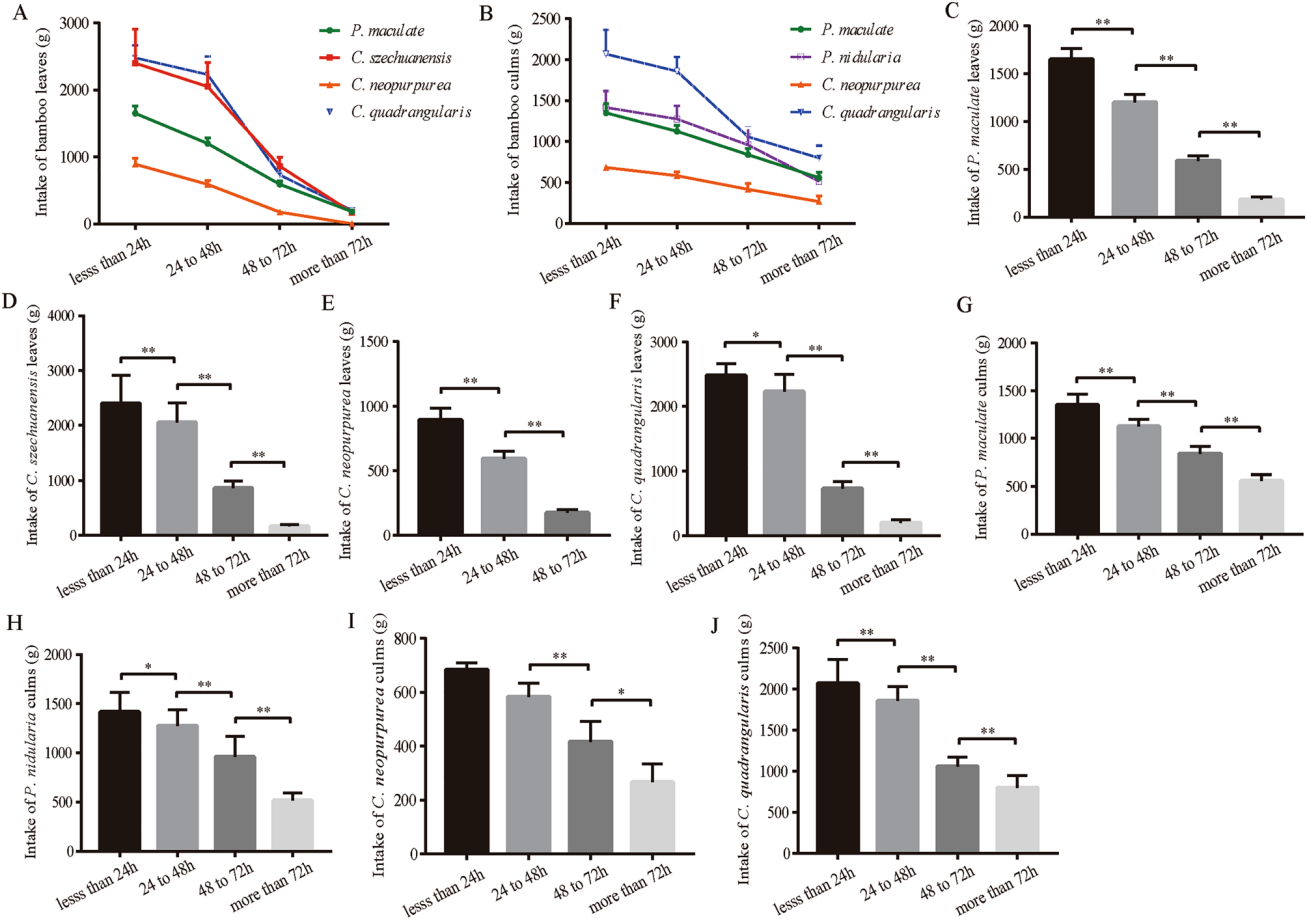
Feed intake of bamboo for captive giant pandas at different feeding time.

To obtain the best felling-feeding time, we set felling-feeding time gradient (less than 24 h, 24 to 48 h, 48 to 72 h and more than 72 h) and analyzed the effect of felling-feeding time on the feed intake of bamboo under the same conditions of other variables, such as the same bamboo age and slope orientation. The results indicated that when felling-feeding time was less than 24 h, the intake of bamboo leaves and bamboo culms was the highest respectively, and a decreased intake was observed with the extension of felling-feeding time. After 72 h, the giant panda hardly ate bamboo leaves at all (Fig. 4A,B).

Next, we analyzed the intake of bamboo leaves and culms in different bamboo species respectively, and found that with the increase of felling-feeding time, the leaves intake of *C. szechuanensis*, *P. maculate*, *C. neopurpurea* and *C. quadrangularis* decreased significantly (*p* < 0.01) (Fig. 4C–F). When felling-feeding time was more than 72 h, the leaves of *C. neopurpurea* were no longer taken (Fig. 4E). Similarly, with the increase of felling-feeding time, the culms intake of *P. maculate*, *P. nidularia*, *C. neopurpurea* and *C. quadrangularis* also decreased significantly (*p* < 0.01) (Fig. 4G–J).

The above results indicated that with the increase of felling-feeding time, a significant decrease was found in bamboo intake, suggesting that we should shorten felling-feeding time and try to maintain the freshness of bamboo.

## Discussion

Bamboo age is one of the factors affecting the feed intake of bamboo. Xie^20^ studied the feeding time distribution of wild giant pandas during the transition period, and found that the feeding time of 1-year-old *Fargesia robusta* leaves was significantly higher than that of perennial bamboo leaves, which was consistent with our results. Although there were slight differences among different bamboo species, the overall trend of bamboo feeding was consistent. Specifically, the 1-year-old bamboo leaves were the most eaten by captive giant pandas, and with the increase of bamboo age, the feed intake decreased, and the 6-year-old bamboo leaves were almost no longer being fed.

Tannin is a kind of secondary compound produced by plants^21^. The feeding taste of animals was reduced by the astringent sensation of tannin, which influenced the feeding behavior of herbivores^22^. Zhao^15^ studied the tannin content of leaves in *Fargesia spathacea* and *Fargesia dracocephala*, and found that the tannin content of leaves of 1-year-old and 2-year-old *Fargesia spathacea* was lower than that of perennial leaves, respectively. However, the tannin content of 1-year-old and 2-year-old *Fargesia dracocephala* leaves was higher than that of perennial leaves, respectively. Therefore, the above results indicate that tannin content of bamboo varies with age.

Previous studies have shown that the nutrient content of bamboo is also affected by bamboo age. Zhao^15^ studied the nutritional composition of bamboo and found that compared with perennial bamboo leaves, annual bamboo leaves had higher protein content. Tang^23^ found that crude protein content of 2-year-old *Bashania spanostachya* culms was higher than that of perennial bamboo culms. The nutrient composition analysis of leaves of *Bashania fargesii* by Wang^24^ showed that crude protein content of 1-year-old bamboo leaves was significantly higher than that of 2-year-old and perennial leaves respectively. Sun^25^ also indicated that crude protein content of *Bashania fargesii* leaves and culms decreased with the increase of bamboo age, and Wang^26^ studied *Bashania fargesii* and found that amino acids content of young leaves was higher than that of old leaves. Therefore, we suspect that the differences in bamboo intake in this study may be related to tannin and nutrient content in different bamboo ages, but further studies are needed to prove this.

Studies have shown that the ecosystem is significantly affected by slope orientation, which is related to vegetation distribution and has a certain impact on soil carbon storage^27–30^. Fan^17^ found that the ecosystem carbon storage of *Phyllostachys edulis* on the sunny slope was significantly larger than that on the shady slope. Wei^31^ suggested that the shoot rate of *Bashania spanostachya* decreased from the sunny slope to the shady slope. Wu^16^ showed that slope orientation significantly affected the height of *Bambusa pervariabilis* × *Dendrocalamopsis grandisi*, in which bamboo on shady slope was 0.5 m higher than bamboo on sunny slope on average. Yang’s^18^ research on natural *Phyllostachys heteroclada* revealed that the bamboo density decreased gradually from the sunny slope to the shady slope. Yu^32^ showed that the chlorophyll value of *Phyllostachys prominens* growing on the sunny side was generally higher than that of bamboo growing on the shady side. Our study demonstrated that feed intake of bamboo growing on the sunny slope was significantly higher than that of bamboo growing on the shady slope. Therefore, we speculate that the differences in bamboo intake may be related to chlorophyll or environmental factors in different slope orientations, but a further study is needed.

In this study, the effect of seasonal, bamboo age, slope orientation and felling-feeding time on bamboo foraging of captive giant pandas were explained, which is helpful to further understand the foraging strategies of captive giant pandas and provide reference for further research on the foraging behavior of giant pandas. At the same time, our study is also helpful to further optimize the diet composition of captive pandas, improve the utilization rate of bamboo, and reduce the economic cost of breeding (According to the internal budget of several panda breeding units, bamboo expenses account for more than 40% of all food expenses, and some units even spend more than 60% on bamboo).

## Methods

### Research site overview

The Dujiangyan Base of China Conservation and Research Center for The Giant Panda is located in Shiqiao Village (103° 33′ E, 34° 57′ N), Qingchengshan Town, Dujiangyan City, Sichuan Province, covering an area of about 51 hectares and 785 m above sea level. The climate here is pleasant, with an average annual temperature is 15.2 degrees Celsius. The average minimum temperature is about 2 degrees Celsius, and the average maximum temperature is only about 28 degrees Celsius. It has abundant rainfall, with an average annual precipitation of about 1300–1800 mm, and a subtropical humid monsoon climate^33^.

### Basic information of captive giant pandas in Dujianyan base

The 14 captive pandas we selected for study are only a portion of the pandas in Dujianyan Base. We strictly follow the relevant provisions of "Technical Regulation of Husbandry and Management of the Giant Panda". In addition to feeding bamboo, we were feeding supplementary food as usual to ensure the nutritional needs of the pandas. The 14 experimental pandas must be healthy, including the following: (1) They have a normal appetite, and can take the initiative to eat bamboo. (2) They are disease-free and their weight meets the standard weight for all ages. (3) They receive regular health check-ups and vaccinations. The basic information of giant pandas is shown in Table 1.

**Table 1:** Basic information about captive giant pandas in Dujiangyan Base.

Name	Stud#	Sex	Birth date	Birth origin	Location	Condition
Yingying	382	Female	1991.9.1	Wolong wild, China	Dujiangyan Base, China	Healthy
Ximeng	399	Male	1993.9.19	Wolong Base, China	Dujiangyan Base, China	Healthy
Yueyue	404	Female	1993.11.20	Wolong Base, China	Dujiangyan Base, China	Healthy
Shulan	407	Female	1994.8.31	Chengdu, China	Dujiangyan Base, China	Healthy
Didi	413	Male	1994.10.5	Wolong Base, China	Dujiangyan Base, China	Healthy
Qiaoyuan	416	Female	1993.9.1	Baoxing wild, China	Dujiangyan Base, China	Healthy
Daili	542	Male	1999.9.1	Baoxing wild, China	Dujiangyan Base, China	Healthy
Taishan	595	Male	2005.7.9	Nzp-wash, USA	Dujiangyan Base, China	Healthy
Hanhan	852	Male	2012.8.21	Yaan Base, China	Dujiangyan Base, China	Healthy
Fubao	887	Male	2013.8.14	Vienna, Austira	Dujiangyan Base, China	Healthy
Bingcheng	938	Male	2014.8.22	Yaan Base, China	Dujiangyan Base, China	Healthy
Jiamei	973	Female	2015.8.15	Yaan Base, China	Dujiangyan Base, China	Healthy
Qingqing	975	Male	2015.8.18	Yaan Base, China	Dujiangyan Base, China	Healthy
Nuannuan	977	Female	2015.8.18	Kualaium, Malaysia	Dujiangyan Base, China	Healthy

### Construction of influencing factors

According to the age division of bamboo culms by Cai et al., the next year of bamboo shoots is one year old, the second year is two years old, and so on. The formula is as follows: the age of bamboo is equal to the year of investigation minus the year of bamboo shoots. The color of bamboo culms, the number of bamboo sheath and the number of leaf sheath are different for different ages of bamboo, so the age of bamboo can be determined by combining these three parameters^34^. Our unit has maintained long-term and stable cooperation with the bamboo planting base located in Chongzhou City, Dujiangyan City and Wenchuan County of Sichuan Province, with a planting area of about 9 square kilometers, and the planting base can provide pandas with a steady supply of fresh bamboo throughout the year. We adopt the method of "Intermediate Cuttings" to collect bamboo. Specifically, according to the feeding needs of the giant panda, we only collect bamboo culms of the same age in the same area. In addition, we collect bamboo leaves of the same age and keep bamboo of other ages so that they can be collected again in the next year.

Slope orientation refers to the direction in which bamboo grows. A sunny slope is a slope that faces due south and always receives sunlight. A shady slope is a slope facing due north, with little or no sunlight. A semi-sunny slope is a slope that faces due south but has a small part of no sunlight, whereas a semi-shady slope is mostly not exposed to sunlight. Our samplers collected bamboo growing in these four different slopes at fixed sites for feeding experiments.

Felling-feeding time refers to the time taken from felling to transporting bamboo to feeding grounds. We transported the felled bamboo to the bamboo storage room in our base, and the storage time was set as less than 24 h, 24–48 h, 48–72 h and more than 72 h. Finally, the bamboo with different storage time was fed at the same time.

### Experimental design and sample collection

In accordance with the feeding sequence of C*. quadrangularis, C. neopurpurea, C. szechuanensis, P. nidularia* and *P. maculate*, the five staple foods of bamboo were fed to all the experimental giant pandas respectively. Leaves of *C. quadrangularis* were fed to all the experimental pandas on the first day of the experiment, and culms of *C. quadrangularis* were fed to all the experimental pandas on the second day, and repeated on the third and fourth days. Leaves of *C. neopurpurea* were fed to all the experimental pandas on the fifth day, and culms of *C. neopurpurea* were fed to all the experimental pandas on the sixth day, and repeated on the seventh and eighth days. In this order, the five staple foods bamboo were fed one by one. All the different factors were controlled so that all the combinations were tested, and each panda received all of the different possible treatments over the course of the experiment.

When studying the factor of bamboo age, leaves of *C. quadrangularis* aged 1 to 6 years old were fed to all the experimental pandas at the same time on the first day of the experiment, and culms of *C. quadrangularis* aged 1 to 6 years old were fed to all the experimental pandas at the same time on the second day, and repeated on the third and fourth day. Leaves of *C. neopurpurea* aged 1 to 6 years old were fed to all the experimental pandas at the same time on the fifth day of the experiment, and culms of *C. neopurpurea* aged 1 to 6 years old were fed to all the experimental pandas at the same time on the sixth day, and repeated on the seventh and eighth days. The five staple foods bamboo aged 1 to 6 years old were fed in this order and all bamboo leaves and culms grew on sunny slopes and felling-feeding time was less than 48 h.

When studying the factor of slope orientation, leaves of *C. quadrangularis* growing in four different slope orientations (sunny slope, semi-sun slope, semi-shady slope and shady slope) were fed to all the experimental pandas at the same time on the first day of the experiment, and culms of *C. quadrangularis* growing in four different slope orientations were fed to all the experimental pandas at the same time on the second day, and repeated on the third and fourth day. Leaves of *C. neopurpurea* growing in four different slope orientations were fed to all the experimental pandas at the same time on the fifth day of the experiment, and culms of *C. neopurpurea* growing in four different slope orientations were fed to all the experimental pandas at the same time on the sixth day, and repeated on the seventh and eighth days. The five staple foods bamboo growing in four different slope orientations were fed in this order and all bamboo leaves and culms were 3 years old and felling-feeding time was less than 48 h.

When studying the factor of felling-feeding time, leaves of *C. quadrangularis* with four different felling-feeding times (less than 24 h, 24 to 48 h, 48 to 72 h and more than 72 h) were fed to all the experimental pandas at the same time on the first day of the experiment, bamboo culms of *C. quadrangularis* with four different felling-feeding times were fed to all the experimental pandas at the same time on the second day, and repeated on the third and fourth days. Leaves of *C. neopurpurea* with four different felling-feeding times were fed to all the experimental pandas at the same time on the fifth day of the experiment, and culms of *C. neopurpurea* with four different felling-feeding times were fed to all the experimental pandas at the same time on the sixth day, and repeated on the seventh and eighth days. The five staple foods bamboo with four different felling-feeding times were fed in this order and all bamboo leaves and culms were 3 years old and grew on sunny slopes.

The experiment began on the 5th, 15th and 25th of each month. Bamboo samples were fed for four consecutive days, and the sample weight of bamboo leaves and bamboo culms is 5000 g, respectively. Bamboo samples were dispersed and placed side by side at 9:00 am, and were recovered at 11:00 am. Then we observed the feeding situation of experimental giant pandas and weighed the remaining unconsumed bamboo.

### Statistical analysis

Bamboo intake refers to the input weight of bamboo minus the remaining unconsumed weight. To make the picture more concise and intuitive, only bamboos eaten by the giant pandas were shown in the results, bamboo species with zero intake were not shown. Excel 2019, GraphPad Prism 7.0 and AI software were used for data analysis and mapping respectively. All data were expressed as mean ± standard deviation (Mean ± SD), where *p* < 0.05 indicates significant difference, *p* < 0.01 means highly significant difference.

### Statement

We confirm that all methods were performed in accordance with the relevant guidelines and regulations of “Technical Regulation of Husbandry and Management of the Giant Panda”, and that the study methods involving plant samples complied with local and national guidelines.

## Supplementary Information

Supplementary Information.

## Data Availability

Our raw data is presented in a supplementary file.
